# Questions for future evidence-informed policy initiatives: insights from the evolution and aspirations of National Immunization Technical Advisory Groups

**DOI:** 10.1186/s12961-020-00551-7

**Published:** 2020-04-22

**Authors:** Anne L. Buffardi, Susan Njambi-Szlapka

**Affiliations:** Overseas Development Institution (ODI), 203 Blackfriars Rd, London, SE1 8NJ United Kingdom

**Keywords:** evidence, policy, health sector, health systems, finance, vaccine, immunisation

## Abstract

**Background:**

Attention to evidence-informed policy has grown; however, efforts to strengthen the quality and use of evidence are not starting from a blank slate. Changes in health architectures and financing pose different considerations for investments in evidence-informed policy than in the past. We identify major trends that have shifted the environment in which health policies are made, and use the evolution and future aspirations of National Immunization Technical Advisory Groups (NITAGs) in low- and middle-income countries to identify questions the sector must confront when determining how best to structure and strengthen evidence-informed health policy.

**Discussion:**

Trends over the last two decades have resulted in a dense arena with many issue-specific groups, discrete initiatives to strengthen evidence-informed policy and increasing responsibility for subnational institutions. Many countries face a shifting resource base, which for some reduces the amount of resources for health. There is global momentum around universal health coverage, reflecting a broader systems approach, but few examples of how the vast array of stakeholders relate within it are available. NITAG aspirations reflect four interconnected themes related to their scope, their integration in national policy processes, health financing and relationships with ministries of finance, and NITAG positioning relative to other domestic and international entities, raising questions such as, What are the bounds of issue-specific groups and their relationship to allocation decision-making processes across health areas? How do technical advisory groups interface with what are inherently political processes? When are finances considered, by whom and how? What is the future of existing groups whose creation was intended to enhance national ownership but who need continued external support to function? When should new entities be created, in what form and with what mandate?

**Conclusions:**

Countries must determine who makes decisions about resources, when, using what criteria, and how to do so in a robust yet efficient way given the existing and future landscape. While answers to these questions are necessarily country specific, they are collective matters that cannot be addressed by specialised groups alone and have implications for new investments in evidence-informed policy.

## Background

Although scholarship on the uptake and use of research dates back decades [1], evidence-informed policy has enjoyed growing attention, both in the health sector and more broadly in international development [2–5]. Evidence can help to identify health problems, better understand their magnitude, causes and consequences, and develop and assess interventions to address these problems; as such, it can help to guide selection among multiple possible courses of action to maximise positive health outcomes or minimise negative effects.

However, efforts to strengthen evidence-informed policy are not starting from a blank slate. Over the last two decades, global initiatives have layered additional structures onto existing domestic architectures. While increased attention and investment in evidence-informed processes holds great potential, like the proliferation of disease- and intervention-specific initiatives, it runs the risk of creating siloed projects that at best operate in parallel to one another, and at worst strain health systems by placing more demands on already stretched staff.

Changes in health architectures, the visibility and organisation of different health focus areas, and financing at national and global levels pose a different set of considerations for investments in evidence-informed policy than at the turn of the millennium. The question, therefore, is not simply, ‘how can the quality and use of evidence on issue X be improved’. We must also ask ‘how do new or renewed efforts to enhance evidence-informed policy relate to existing, and in some cases, shifting structures and resources?’ Here, we use the evolution and future aspirations of National Immunization Technical Advisory Groups (NITAGs) in low- and middle-income countries (LMICs) – groups of national experts who provide evidence-informed recommendations to ministries of health – to illustrate four core questions and tensions that the sector must confront when determining how best to structure and strengthen evidence-informed health policy. These interconnected questions relate to the scope of groups’ mandate, their integration in national policy processes, health financing and relationships with the Ministry of Finance, and the position of issue-specific groups relative to other domestic and international entities.

### Previous efforts to strengthen evidence-informed health policy

NITAGs represent one type of numerous efforts to enhance evidence-informed health policy, which we briefly review in order to situate NITAGs within the broader field. For many decades, universities and other institutions have participated in longstanding cross-national exchanges, established twinning arrangements and organised study tours to share knowledge and strengthen capacity. In addition to efforts to improve the availability and quality of evidence, more recent initiatives have aimed to enhance the demand for and capacity to interpret and apply evidence, with a more explicit focus on decision-makers and facilitating interactions between knowledge producers (researchers) and users (policy-makers)^1^ [6].

Initiatives have run the gamut in terms of scope. They range from investments to support evidence in a particular issue area in one country, to multi-country health initiatives targeting specific population groups like the Innovating for Maternal and Child Health in Africa initiative [7], to multi-country initiatives aimed at the sector as a whole, like the WHO Alliance for Health Policy and Systems Research programme Sponsoring National Processes for Evidence-informed Policy in the Health Sector of Developing Countries [8] and the EU-funded project Supporting the Use of Research Evidence (SURE) for Policy in African Health Systems [9, 10] . Glassman and Chalkidou identified 28 distinct international initiatives supporting resource prioritisation efforts in the health sector in LMICs, a number which has likely grown since initially estimated in 2012 [11].

Similarly, research councils have funded cross-national investigation of evidence use in LMICs. This work has been focused on specific areas like sexual and reproductive health, HIV [12], eclampsia and malaria [13] as well as studies looking across a much wider range of health areas and country income groups [5]. Bilateral initiatives have invested in evidence-informed policy more broadly, including but not limited to the health sector; for example, the Knowledge Sector Initiative in Indonesia [14, 15] and the Building Capacity for the Uptake of Research Evidence (BCURE) programme, which worked in a dozen countries in Africa and Asia [16]. Together, these initiatives highlight the importance of the perceived relevance of the evidence, regular interactions between researchers and policy-makers to build and maintain relationships (made more challenging by frequent turnover of ministry staff), and attention to institutional procedures and incentives as well as individual capacities and commitment.

NITAGs represent a specific type of evidence-informed decision-making body, which are currently functioning in 123 countries^2^ [18]. Although they have existed in some countries since the 1960s, their establishment and development in LMICs was bolstered by the Supporting Independent Immunization and Vaccine Advisory Committees (SIVAC) initiative; from 2008 to 2017, this Gates-funded initiative supported NITAGs in 43 countries [19–22]. NITAGs are formal groups with a legislative or administrative basis, comprised of national experts who provide evidence-informed recommendations on vaccine-related issues. Members serve in an individual capacity, rather than as representatives of institutions, and typically have immunisation-related disciplinary backgrounds like paediatrics, infectious disease, epidemiology, immunology and, less commonly, health economics. According to NITAG guidance materials, they should declare conflicts of interest, have formal terms of reference and distribute agenda items prior to meetings in order to facilitate transparent processes and independent advice [17, 23]. Members’ time is offered in-kind; secretariat support, meeting and other costs are intended to be funded through the national ministries of health, but many NITAGs continue to require external resources to function [22, 24]. Relative to the initiatives described above, NITAGs place more attention to the types of evidence used and procedures for the groups’ operation, including how evidence is gathered and analysed and how policy recommendations are reached, rather than external communication strategies or processes for interacting with other decision-making bodies.

Previous research on NITAGs and other efforts to strengthen evidence-informed policy has characterised ways of working [25–28], compared differences across national contexts [5, 12, 13, 20, 29–33], and retrospectively evaluated the effectiveness of specific programmes [9, 16, 21, 22, 34]. A recent evaluation of NITAGs in LMICs indicated that NITAGs were perceived as valuable instruments of nationally owned decision-making, which drew on local evidence when possible and could tailor global and regional recommendations to national contexts. Although their capacity to gather and use evidence varied, and newly established NITAGs were thought to need continued financial and technical support, considerable progress was noted in the establishment of these bodies as formalised, transparent means of using evidence to determine recommendations regarding national vaccine decisions [22, 24, 34].

This article aims to be forward looking, using NITAG aspirations and an analysis of the context in which they increasingly have to operate to highlight questions that similar groups may face. However, many of these questions and challenges cannot be addressed by individual groups on their own – these are collective issues that the sector as a whole must confront.

These analyses draw on primary and secondary source information gathered as part of a larger project on NITAG decision-making and support options, which involved document review, outputs from a NITAG stakeholder workshop and interviews with key informants from 14 countries [35]. Documents included articles from the academic and grey literature, NITAG evaluations, presentations, standard operating procedures, meeting minutes, recommendations and other national NITAG materials. The countries covered five WHO regions as well as a range of income levels and Gavi eligibility and transitioning status. The median NITAG founding date was 2010 (range from mid-1960s to 2015), so this set of countries may reflect different experiences than those with very recently established groups. Moreover, the vast majority of key informants were directly affiliated with NITAGs, so their perspectives do not represent the full range of stakeholders in each country; further dialogue on the questions raised in this article would benefit from involvement of a wider set of actors.

### Overarching trends shaping national health policy processes

Four major trends over the last 20 years have shifted the environment in which health policy decisions are made, namely (1) the expansion of the health and development agenda and proliferation of formalised groups, many of which are disease or intervention specific; (2) the recent swing-back towards more of a systems perspective; (3) a shifting resource base as national incomes rise and international donors withdraw; and (4) greater targeting at subnational levels, also with implications for the number of entities involved in health policy.

Over the last two decades, the number of national-level committees and advisory groups and global health networks has multiplied – a trend which has been widely acknowledged [36–38]. Many of these groups are specialised, with a focus on particular diseases, population groups or interventions. The number of NITAGs doubled from 2010 to 2016 [39] and the Global Vaccine Action Plan calls for all countries to have access to such a group by 2020 [40]. In some cases, like with NITAGs in many LMICs, the creation of new entities was driven by global strategies and the availability of external funding. Other groups were mandated by global health initiatives, like Gavi Inter-agency Coordinating Committees and Global Fund to Fight AIDS, Tuberculosis and Malaria (GFATM) Country Coordinating Mechanisms [41, 42]. Some of the research-to-policy initiatives mentioned in the introduction also sought to create new networks and groups, like Health Policy Committees in Nigeria [43].

The intent with many of these entities was to encourage national ownership and foster more inclusive processes; for example, directly involving people living with HIV in Country Coordinating Mechanisms, bringing together multiple disciplinary specialisations to advise on immunisation policy, and promoting connections between researchers and policy-makers. In some instances, the creation of such groups also explicitly aimed to advance the use of evidence in health policy. Although infectious disease communities have often been first movers, other health areas have (re)organised themselves to raise attention to newborn, maternal and child health [44] and non-communicable diseases [45], among others, increasing the number of constituencies at global and national levels.

The Sustainable Development Goals (SDGs) reflect this more expansive agenda, both with regards to the number of areas included within the health goal as well as the full set of issue areas covered. The centrality of universal health coverage (UHC) within the health SDGs also marks a swing back towards a systems approach. This represents a shift away from the vertical orientation of the major global health initiatives of the previous decade – in terms of normative commitments if not (yet) in terms of development financing for health [46]. Scholars, and more recently the WHO Strategic Advisory Group of Experts on Immunization, have acknowledged the need to integrate immunisation efforts within a broader system-wide approach linked to UHC and the SDGs [39, 47, 48]. However, to date, research has predominantly highlighted missed opportunities and the difficulties in attempts at integration [49–53]. The WHO has initiated discussions about the use of health technology assessment (HTA) within the context of UHC as a policy tool for systematically evaluating the properties, effects and impacts of health interventions. This could serve an integrating function, with the purpose of allocating finite resources and improving equitable access [54]. However, HTA, particularly through formalised entities, remains uncommon in LMICs [55–57].

As the set of issues that governments are expected to address has expanded, a growing number of countries are transitioning away from external support, leaving some with a more restricted resource base with which to address these needs. For example, 35 low-income countries have been reclassified as middle-income countries in the last 15 years. Another 29 countries are expected to be ineligible for official development assistance by 2030 [58]. In the last several years, 16 countries have become ineligible for Gavi assistance, with another 26–31 projected to be fully transitioned by 2025 [59]. The departure of multiple global health initiatives within a short period of time represents a substantial reduction in health financing for some countries in particular, including multiple fragile and conflict-affected states and populous countries like Nigeria and Pakistan [60]. Furthermore, recent analyses indicate that, as country incomes have risen, sectoral resource allocation, including both government spending as a proportion of gross domestic product as well as official development finance, has shifted away from health and education towards infrastructure, further limiting investments in health [58].

A final trend worth noting are moves toward greater geographic specialisation and targeting. A number of countries, including Kenya, Nepal and Indonesia, have initiated processes of decentralisation. To varying degrees across countries, these changes devolve responsibility for healthcare delivery, decision-making and resource allocation to subnational levels. Even for countries that are not in the midst of decentralisation, there is growing recognition of the importance of targeting key populations [61] and subregions in greatest need [62]. Like disease-specific coordinating and advisory groups, these geographic shifts increase the number of entities involved in the decision-making and implementation chain. These last two trends affect some countries much more than others and, for some, they are in the early stages and therefore their consequences may take time to be observed.

Taken together, these trends have resulted in a dense arena with many existing bodies, including issue-specific groups, discrete initiatives intended to strengthen evidence-informed policy and increasing responsibility for subnational institutions; a shifting resource base which, for some countries, is reducing the amount of resources available for health, including investments in the quality and application of evidence; and global momentum around UHC, reflecting a broader systems approach, but few examples of how the vast array of stakeholders relate within it. We now turn to the experiences of NITAGs in selected LMICs to illustrate how this confluence of factors is manifesting itself in practice through these formalised evidence-informed advisory groups.

### NITAG aspirations and questions

When NITAG members and other stakeholders were asked where they envisioned NITAGs in the future, four interrelated themes emerged related to the scope of their mandate, integration in national policy processes, health financing, and their position relative to other domestic and international entities. First, NITAGs sought a more proactive role that covered additional topics – but one that remained within the remit of immunisation. Many saw an opportunity to broaden their scope beyond review and recommendation of new vaccines. More recently established NITAGs have typically responded to requests from the Ministry of Health and expressed a desire to also identify relevant issues themselves to raise with the ministry. They saw a need to expand their focus beyond childhood vaccinations to consider immunisation issues in other age groups. Others mentioned the possibility of advising on outbreak response and implementation matters, including modifications to the vaccine schedule and coverage rates. Consideration of these topics takes NITAGs beyond upstream decisions about new vaccine introduction to involvement in implementation and evaluation phases of the policy cycle.

That said, NITAGs did not raise the possibility of extending their mandates beyond immunisation.^3^ With one exception, informants did not discuss how NITAG recommendations fed into broader assessment processes which weighed vaccine investments against other health interventions or how the group related to decisions about the composition of a national health benefit package. These interviewees did not independently mention HTA and, when asked directly, were not familiar with the process, including in a country that had been initiating more formalised HTA. The experience in one country with an active HTA body diverged from the overall trend; in this instance, the NITAG’s mandate had shifted and an additional entity was being created to coordinate the country’s multiple advisory and decision-making groups.

Thus, when looking towards the future, NITAGs appeared to be eager to broaden their scope within immunisation but not beyond it. These perspectives raise the question, what should be the bounds of issue-specific advisory groups and their relationship to broader sector-wide efforts that involve policy and resource decisions across multiple health areas and interventions?

Second, NITAGs wanted to become more integrated in national policy processes, reflecting a core theme and frequent challenge of initiatives to strengthen evidence-informed policy. Greater integration serves the purpose of streamlining efforts, better linking evidence generation and assessment to specific policy needs. It was also perceived to enhance the influence of the NITAG, increasing the likelihood that their recommendations would be adopted and implemented.

Our informants did not use evidence-informed policy jargon like research uptake or demand for evidence, but saw requests for their advice, the approval and implementation of their recommendations and, ultimately, improvements in health outcomes as key indicators of their success. As noted, a recent evaluation indicated that ministries of health in case study countries valued NITAG contributions [22, 34]. However, several interviewees from newer NITAGs were unclear of what happened, if anything, once a recommendation had been issued, if it was disseminated within the ministry to all of the relevant people, and were uncertain if further action was being taken.

The initial request from the Ministry of Health for NITAG advice was often issued through formal means. As the decision-making process progressed, it became less well defined. This may be due, in part, to the iterative nature of policy processes, where further information requests and discussions took place through a series of interactions with different people and agencies.

On the one hand, integration of specialised advisory groups into health policy processes involves some practical and logistical matters, particularly when there are a number of groups who may want to be more proactive and who are not linked to one another. When, how and with whom should each group interact? Countries with decentralised health systems must ask this question at the subnational level as well, where processes may vary across districts and the number of potential interaction points multiplies significantly. This was not yet an issue for NITAGs. Those with whom we spoke were only involved in processes at the national level at that time. One country was undergoing a substantive devolution process and the implications for the NITAG were unclear at that stage. This interviewee wondered where the approval of future NITAG recommendations would take place (at national and/or subnational levels) and whether or not the composition of the NITAG would need to change to include representatives from each province. In another country, provinces became responsible for the financing and delivery of vaccines after the first 2 years of national rollout. The NITAG was only involved in the initial review and recommendation phase with the Ministry of Health, although subnational decisions and activities directly affected vaccine coverage rates – an outcome of interest for NITAGs.

Determining where and when evidence-informed advice feeds into iterative processes is one question; however, integration into policy processes involves more than matters of organisation and coordination. As NITAGs seek to become more embedded, it raises the question of how do technical advisory groups interface with what are inherently political processes?

This question was never explicitly articulated as such by our informants and previous research on NITAGs has discussed the issue of NITAG integration in rather abstract ways. Articles have indicated the need for NITAGs to balance integration and independence [22], and for their “*careful positioning*” in decision-making processes [20]. Key informants did note the interpersonal element of policy processes and the importance of establishing and maintaining relationships with people in relevant (and sometimes frequently changing) decision-making positions. They aimed to be perceived as credible advisors, based on their ability to marshal pertinent and rigorous evidence and communicate it effectively, including in non-technical ways to audiences who were not experts in the subject.

On the whole, NITAG experiences thus far are better characterised by the lack of contestation rather than the dominance of it. In one instance, another assessment body disagreed with the NITAG because of cost considerations, but overt disputes appeared to be uncommon. When evaluating clinical and epidemiological evidence, there may be a clear case of the public health benefits of vaccination on which evidence-based technical advice can be given. As vaccines, particularly newer, more expensive formulations, are no longer externally co-financed by Gavi, and they are weighed against other public priorities within and beyond the health sector, the political nature of these processes will become more apparent. The extent to which evidence is valued, NITAG recommendations are considered by final decision-makers and health outcomes are improved is out of NITAGs’ direct control, which may limit their ability to fully achieve the aims they seek. It also has implications for their ways of working and profiles of members, which, to date, has emphasised technical rather than relational and influencing skills.

Indeed, financing, and the role of the Ministry of Finance, was recognised as playing a highly influential role regarding if, when and how NITAG recommendations were adopted and implemented. Although there were no examples of NITAG recommendations being rejected outright by ministries of health, informants mentioned modifications and delays in implementation, in some cases for many years. Some NITAGs indicated that, during their deliberations, NITAG members were broadly aware of what would be financially feasible, although this was often not part of formal assessments. In recent years, there has been increasing recognition of the need to integrate economic analyses [24]. Most NITAGs did not interact directly with ministries of finance; these negotiations were predominantly led by ministries of health. One interviewee noted the importance of timing and questioned whether recommending vaccine adoption prior to determining the price undermined countries’ negotiating power with manufacturers, rather than making recommendations contingent upon a feasible financial agreement. The withdrawal of substantial sources of international funding, coupled with financial devolution in some countries, may further complicate the already difficult task of accurately forecasting national and subnational budgets and identifying what is financially possible.

Questions about financing relate to both of the previous sets of questions around NITAG scope and integration. As resource prioritisation becomes a more pressing concern, at what point in the policy process are finances considered, by whom and how? Should financing be considered as an initial binding constraint, as a matter of negotiation within a budget range, or as a second stage assessment following technical recommendations about intervention effectiveness? What is the role of advisory groups and line ministries in these discussions?

The final set of questions that NITAG experiences raise relates to the relationship of this issue-specific advisory group to other domestic and international actors beyond government ministries. Despite the presence of numerous other groups in the health sector noted earlier, NITAGs did not have formalised relationships with other entities. NITAG members often had academic affiliations and were involved in professional associations, so could facilitate linkages with these organisations. This took place on an individual rather than institutional basis, potentially risking the loss of important relationships as members change over time. NITAGs, Gavi Inter-agency Coordinating Committees and other vaccine advisory groups did not interact with one another and the presence of multiple groups created confusion at the national level [22, 30, 34]. Thus, there appears to be fragmentation among entities even within the same health focus area.

In contrast to the lack of formal ties and clear relationships with other domestic entities, many newer NITAGs, particularly in lower income countries, have been financially dependent on international donors. Recent assessments indicate that they will continue to need this support for at least another decade [22, 24]. As has been reported elsewhere, Gavi has played an influential role in national decision-making, in some cases bypassing NITAGs [22, 30, 34].

NITAG relationships with other domestic and international organisations raise several questions: how could established groups with sustainable sources of funding better coordinate with one another and other stakeholders they share in common (principally the Ministry of Health)? What are feasible options for existing groups that aim to enhance national ownership, whose creation was largely externally driven by global strategies and international financing, who continue to need external financial support in order to function? The situation is different for countries without specialised groups, for example, in the 71 countries without a functioning NITAG. In these cases, how can evidence and advice on particular technical and health areas inform national health policy when specialised groups do not exist? When should new groups be created, in what form (i.e. enduring entities, time bound task forces, ad hoc working groups), and with what mandate or scope?

## Discussion

As portrayed above, within the wider context of an expanded health agenda, dense arena, and donor transitions, NITAG aspirations indicate the desire for an expanded and more integrated role, the bounds of which are not yet fully defined. Topically, they see themselves grounded in the vaccine space, as the group name and member expertise reflect. However, if they become more involved in policy processes, including financing and resource allocation decisions, and interact more with government ministries and other committees and advisory groups, this will start to shift their highly technocratic, issue-specific orientation. The specific mechanisms for these increased interactions, too, are largely undefined, although UHC and resource prioritisation efforts may offer an overarching organising structure.

To varying degrees, NITAG aspirations reflect all four of the broader trends highlighted here, although they may not explicitly be perceived as such. They anticipate the implications of shifts that are still at early stages, including more of a systems orientation and departure of some international donors. Decentralisation and more specialised targeting do not appear to have affected NITAG mandates to date, although they may in the future, and countries for whom this is most relevant are cognisant of this potential eventuality.

There remains a fundamental tension for some NITAGs and other groups that are intended to be nationally owned but who are financially dependent on external donors to function. For countries most affected by donor withdrawals, their more limited resources may result in a period of contraction, both in terms of the number of groups and the breadth of their mandate. Some committees may fold or be merged into other entities. These changes may leave deficits or an imbalance between a country’s disease profile and the specialisations of the groups remaining. Evidence can help to guide these resource prioritisation efforts, both in terms of allocation across an expansive set of national needs as well as regarding which advisory processes are best suited to inform these decisions. At a minimum, the shift back towards a systems orientation will likely require issue-specific groups to broaden their perspective, if not their mandate, and to more intentionally consider how they fit within the overall health landscape; this could improve coordination and may also induce territorial responses and competition.

The health sector has long debated the comparative advantages of specialisation versus breadth, and highlighted concerns regarding fragmentation and coordination among many actors [49, 63–67]. These discussions have been less prominent in the broader field of evidence-informed policy. The latter, however, has more extensively examined the political aspects of policy processes, which the health sector has been critiqued for minimising [68–70]. NITAG aspirations and challenges touch on all of these aspects and so could benefit from and contribute to these somewhat parallel scholar and practitioner communities.

## Conclusions

The set of questions posed here suggest a redefining of scope and bounds, the specific details of which will necessarily be country-specific and evolve over time. Notably, these are not matters that NITAGs, other specialised groups or discrete initiatives to strengthen evidence-informed policy can address on their own. We articulate these questions here to enable health sector actors to consider these questions and trade-offs in a more deliberate and proactive way, and to do so in a joined up rather than siloed fashion. Some of these questions relate to longstanding, largely unresolved challenges, like issues of coordination and the role and implications of international donors in national decision-making. Other questions are considerations that groups are just beginning to or anticipate having to confront, including increased attention to domestic resource prioritisation, interactions with ministries of finance and subnational actors, and potential involvement in more downstream phases of the policy process.

Domestic and international entities are implicitly experimenting with different ways of supporting evidence-informed policy. Examining how different countries and configurations of groups within them respond to these questions as they adapt to broader trends would be a rich line of future enquiry. More practically, NITAG experiences highlight two considerations for future efforts to strengthen evidence-informed policy. First, as noted in the introduction, is the importance of acknowledging existing and unfolding processes, structures and resource shifts when initiating new programmes. The second relates to the content and focus of such initiatives.

Capacity-building and facilitating interactions between knowledge producers and knowledge users have often been core components of previous efforts to strengthen evidence-informed policy [6]. These strategies will likely remain relevant, but the types of capacity needs and interactions may be more varied. NITAG experiences highlight a gap in economic analysis and an increased orientation towards more relational capacities as they seek to further integrate into policy processes. The latter represents a departure from their strongly technical orientation and emphasis of previous support on the generation and assessment of evidence. Similarly, future efforts to facilitate interactions may involve different profiles of people and institutions, including relationships between line and finance ministries, who have been much less of a focus in the past, and relationships among multiple issue-specific advisory groups who both produce and use evidence.

Fundamentally, countries are faced with the task of determining who makes decisions about resource investments, when, considering what basket of alternatives, and according to what criteria or standards. How to do so in a transparent, robust, yet efficient way given the existing, and in some cases changing, institutional architecture at global, national and subnational levels represents a key challenge in the coming years. There is an opportunity to approach this challenge in an intentional, collective manner, guided in part by emerging questions such as those posed here.

## Data Availability

The full list of documents reviewed and key informant interview guides are available from the corresponding author on reasonable request.

## Abbreviations

HTA: health technology assessment
LMICs: low- and middle-income countries
NITAG: National Immunization Technical Advisory Group
SDG: Sustainable Development Goal
UHC: universal health coverage

## Footnotes

1 For example, these initiatives sought to build capacity of researchers to present evidence in ways that are more accessible to policy-makers like research syntheses and policy briefs; provided training, technical support to develop tools and guidelines, accompaniment and mentoring to policy-makers to apply evidence; supported intermediary organisations, including the media, civil society and specialised functions within research institutes, to serve as knowledge brokers; established rapid response facilities to address pressing evidence needs of policy-makers; and created mechanisms to increase communication between researchers and policy-makers such as structured meetings, informal luncheons and deliberative dialogues.

2 The WHO defines a functional NITAG as meeting all six of the following process indicators: legislative or administrative basis for the advisory group, formal written terms of reference, at least five different areas of expertise represented among core members, at least one meeting per year, circulation of the agenda and background documents at least 1 week prior to meetings, mandatory disclosure of any conflict of interest [17]. An additional 48 countries report a NITAG that meet between one to five of these criteria.

3 One longstanding NITAG with whom we spoke was quite distinct from the others in that it advised on communicable diseases rather than solely immunisation matters, was structurally embedded within the Ministry of Health and had a much larger membership. They did not indicate a desire to expand their already broad mandate.
